# Reply to the Letter to the Editor by C. Nicolazzo et al.: “Circulating Cell-Free DNA and Circulating Tumor Cells as Prognostic and Predictive Biomarkers in Advanced Non-Small Cell Lung Cancer Patients Treated with First-Line Chemotherapy”

**DOI:** 10.3390/ijms18061309

**Published:** 2017-06-19

**Authors:** Angela Alama, Simona Coco, Irene Vanni, Francesco Grossi

**Affiliations:** Lung Cancer Unit, IRCCS AOU San Martino—IST Istituto Nazionale per la Ricerca sul Cancro, L.go R. Benzi 10, 16132 Genova, Italy; simona.coco@hsanmartino.it (S.C.); irene.vanni@hsanmartino.it (I.V.); francesco.grossi@hsanmartino.it (F.G.)

## Reply:

Thank you for the valuable comments. We agree with the concern that distinguishing genuine circulating tumor cells (CTCs) from other circulating cells by morphology may be questionable. Currently, the CellSearch isolation system (Janssen Diagnostics, Raritan, NJ, USA) is the only Food and Drug Administration (FDA) approved methodology to identify CTCs in breast, prostate and colon cancers. [1,2]. However, immunophenotyping with pan-cytokeratins and CD45 (for leukocytes) cannot ensure the certain nature of neoplastic elements shed from primary or metastatic tumors into the bloodstream. Indeed, circulating cells with epithelial-to-mesenchymal transition phenotypes (reduced or absent epithelial cell adhesion molecule (EpCAM) expression) are often missed by this technique and can be underestimated [3]. For the same reason, the characterization of CTCs solely using a panel of cytokeratins in non-small-cell lung cancer (NSCLC) remains a matter of debate [4] and, to the best of our knowledge, data on CTC expressing thyroid transcription factor 1 (TTF-1) in NSCLC are rare and not conclusive [5]. In the attempt to overcome this controversial issue, we referred to the isolated non-hematologic circulating cells with malignant features in our study. These cells were morphologically identified and enumerated according to the criteria described by Wechsler [6] excluding clearly distinguishable contaminant leukocytes and ambiguous elements such as circulating giant macrophages, monocytes and megakaryocytes, if present. 

In a preliminary explorative study, we investigated the presence of suspicious malignant elements in the peripheral blood of cancer patients and healthy individuals by morphology associated to immunofluorescence for 4',6-diamidino-2-phenylindole (DAPI) and cytokeratin 7 (CK7). Moreover, in a collaboration study with Bozzetti et al. [7], a non-EpCAM-based capture method was performed to isolate and characterize suspicious malignant elements in the peripheral blood of cancer patients and healthy subjects. Unexpectedly, after depletion of leukocytes and erythroid cells, suspicious circulating elements were also found in healthy individuals [7]. 

As correctly suggested, additional molecular characterizations of isolated non-hematologic circulating cells with malignant features would help support our results. However, immunocytochemical and/or molecular investigations are not always feasible for the low presence of suspicious cells requiring reliable challenging procedures to confirm their malignant origin. 
